# Acquisition of the Conjugative Virulence Plasmid From a CG23 Hypervirulent *Klebsiella pneumoniae* Strain Enhances Bacterial Virulence

**DOI:** 10.3389/fcimb.2021.752011

**Published:** 2021-09-09

**Authors:** Dongxing Tian, Weiwen Wang, Meng Li, Wenjie Chen, Ying Zhou, Yunkun Huang, Zike Sheng, Xiaofei Jiang

**Affiliations:** ^1^Department of Clinical Laboratory, Huashan Hospital of Fudan University, Shanghai, China; ^2^Department of Clinical Laboratory, The First Affiliated Hospital of Guangxi Medical University, Nanning, Guangxi, China; ^3^Department of Infectious Disease, Huashan Hospital of Fudan University, Shanghai, China; ^4^Department of Clinical Laboratory, Shanghai Pulmonary Hospital of Tongji University, Shanghai, China; ^5^Department of Clinical Laboratory, Kunming Yan’an Hospital, Kunming, China; ^6^Department of Infectious Diseases, Ruijin Hospital, Shanghai Jiao Tong University School of Medicine, Shanghai, China

**Keywords:** conjugative, transfer, dissemination, plasmid, siderophore production, hypervirulence, hypervirulent *Klebsiella pneumoniae*

## Abstract

The emergence of hypervirulent and carbapenem-resistant *Klebsiella pneumoniae* (hv-CRKP) has become a hot topic and confounding problem for clinicians and researchers alike. Conjugative virulence plasmids have the potential to cause more threatening dissemination of hv-CRKP strains. We previously identified K2606, a CG23 clinical hypervirulent strain of *Klebsiella pneumoniae* harboring a conjugative virulence plasmid designated pK2606. In this study we examined hypervirulence levels using assays of biofilm formation, serum resistance, and wax larvae and mouse *in vivo* infection models. Moreover, to define the transfer ability of pK2606 and whether this confers hypervirulence to other strains we performed plasmid transconjugation experiments between K2606 and the ST11 CRKP strain HS11286 along with *E. coli* J53. We found that although biofilm formation and serum resistance were not significantly increased, the transconjugants acquired the ability of produce high level of siderophores and also caused high mortality of wax larvae and mice. Furthermore, we identified pK2606-like conjugative virulence plasmids in GenBank, providing evidence that such plasmids may have begun to spread throughout China. These findings provide an evidence base for the possible mechanisms of the emergence of hv-CRKP strains and highlight the potential of pK2606-like conjugative virulence plasmids to spread worldwide.

## Introduction

*Klebsiella pneumoniae* is considered as a major threat to public health. This microorganism is not only cause severe invasive disease that affect healthy people in the community, but also has a significant propensity to acquire drug resistance in the nosocomial setting (Navon-Venezia et al., 2017; Harada and Doi, 2018). However, while strains rarely possess both of these properties, there are increasing reports of multidrug-resistant and hypervirulent strains (Wyres et al., 2019; Zhang et al., 2020). Traditionally, hypervirulent strains have only been related with some particular types, such as the capsular type K1 sequence type 23 (K1-ST23) and K2-ST86 (Bialek-Davenet et al., 2014; Lam et al., 2018a), which are commonly susceptible to antibiotics. More recently, virulence clusters have also been identified in other STs which are always associated with resistance, such as ST11 and ST15 (Gu et al., 2018; Shu et al., 2019). These virulence clusters, including iron acquisition regulatory genes (especially aerobactin, but also salmochelin) and regulators of mucoid phenotype A (*rmpA*) and *rmpA2*, are carried on a large virulence plasmid.

The emergence of hypervirulent and carbapenem-resistant *Klebsiella pneumoniae* has become a hot topic, and research is increasingly focused on the mechanism of transmission, but this currently remains unclear. The virulence plasmids acquired by ST11 carbapenem-resistant *Klebsiella pneumoniae* (CRKP) are commonly pK2044-like plasmids, which are considered non-conjugative (Smillie et al., 2010; Tian et al., 2021). According to several studies these virulence plasmids confer hypervirulence to other strains (Gu et al., 2018; Zhang et al., 2020). Although uncommon, virulence plasmids with complete Type IV Secretion Systems (T4SSs) must also not be overlooked. Conjugative virulence plasmids have the potential to transfer to other resistant strains or species, endowing them with a hypervirulent and resistant phenotype. Due to their self-conjugation ability, the encoded critical virulence factors of conjugative virulence plasmids, especially the aerobactin genes *iucABCD-iutA*, will be transferred along with the plasmid. This combination of hypervirulence and resistance poses a serious challenge to both clinicians and researchers. Indeed, once conjugative virulence plasmids are transferred among different strains in the hospital setting, the situation becomes even more intractable.

Our earlier study provided a scheme to distinguish conjugative virulence plasmids from non-conjugative virulence plasmids by assessment of IncFIB_K_ genetic diversity (Tian et al., 2021). However, the virulence level conferred by conjugative virulence plasmids needs to be further characterized. CG23 is considered to be highly related to conserved hvKp strains that harbor pK2044-like virulence plasmids (Lam et al., 2018b), whereas conjugative virulence plasmids are possibly associated with other clonal groups. We recently identified a CG23 clinical strain K2606 from a patient with bloodstream infections. K2606 harbored a conjugative virulence plasmid, while no pK2044-like virulence plasmid was found in it. Nevertheless, the virulence of the K2606 strain and transfer ability of pK2606 plasmid need to be determined. In particular, it also remains urgent to confirm whether such conjugative virulence plasmids confer hypervirulence to other strains.

## Materials and Methods

### Strains and Growth Conditions

*K. pneumoniae* strain K2606 was isolated from blood in 2012 at Huashan Hospital (Shanghai, China), and were sequenced completely (GCA_011006575.1). Carbapenem-resistant *Klebsiella pneumoniae* HS11286 was isolated from sputum in 2011 at Huashan Hospital (Shanghai, China) and were sequenced completely before (GCA_000240185.2) (Liu et al., 2012). All strains were stored in 20% (w/v) glycerol at −80°C and cultured on Luria Broth medium at 37°C. Plasmid stability experiments were performed as described previously (Zhou et al., 2020). For LB growth experiments, the bacteria was grown at a starting A_600_ of 0.05 in LB at 37°C, and the A_600_ was measured each hour. Each curve was performed in triplicate.

### Antimicrobial Susceptibility Test

The antimicrobial susceptibility was performed using the microdilution method and interpreted according to the Clinical and Laboratory Standards Institute (CLSI, 2020). Antimicrobial agents tested included amikacin, ampicillin/sulbactam, piperacillin/tazobactam, cefazolin, cefuroxime, ceftriaxone, cefepime, chloramphenicol, gentamicin, levofloxacin, trimethoprim/sulphamethoxazole, meropenem, tigecycline, and nitrofurantion. 

### Conjugation Assay

Carbapenem-resistant *K. pneumoniae* HS11286 and sodium azide-resistant *E. coli* J53 were used as a recipient, and *K. pneumoniae* K2606 strain was used as the donor. The kanamycin-resistant gene was introduced into pK2606 by λ-Red-mediated homologous recombination method to be selected as the donor resistance marker. The primers were included in **Table S3**. Both donors and recipients were cultured to logarithmic phase at 37°C, and 200ul of donor cells and 800ul of recipient cells were mixed and inoculated on the LB agar plate at 37°C overnight. Next, the transconjugants were selected with the meropenem (1ug/ml) and hygromycin (400ug/ml) if HS11286 was used as a recipient, or sodium azide (150ug/ml) and hygromycin (400ug/ml) if *E. coli* J53 was used as a recipient. The transconjugants was determined by PCR using *iucA, oqxA*, and *ICE* as marker genes. PFGE, S1-PFGE, and plasmids draft sequencing were performed as described previously (Pecora et al., 2015; Tian et al., 2020). Sequences were submitted to GenBank (MZ475693, MZ475694, MZ475695, MZ475696, MZ475699, MZ475705, MZ475706, MZ475707, and MZ475708).

### Biofilm Formation Assay

Biofilm formation was performed as described previously in 96-well microtiter plates (Choi and Ko, 2015). Briefly, 200μl of the mid-log phase bacteria cells (1.5x10^7^ CFU/ml) was added to 96-well microtiter plates and incubated overnight. Next, all cultures were removed and then washed twice with phosphate-buffered saline. 0.1% crystal violet solution was used for staining for 20 min and washed three times. The biofilm formation was quantified by measuring the A_590_ after solubilized with 200 μl of 95% ethanol. Each assay was performed in duplicate and repeated three times independently.

### Serum Resistance Assay

The serum resistance was determined according to a previous study (Choi and Ko, 2015). The mid-log phase bacteria cells were mixed with normal human serum at a 1:3 ratios and then incubated at 37°C for 2h. Serum resistance were characterized by plotting the average survival percentage of each strain against the incubation time. All assays were performed three times independently. *K. pneumoniae* strains were considered resistant to normal human serum if at least 90% of the organisms survived and were considered sensitive to serum if CFU counts dropped to 1% after 2 h of incubation.

### Greater Wax Moth Larvae and Mouse Infection Models

*G. mellonella* larvae weighing about 300 mg, which were purchased from the Tianjin Huiyude Biotech Company, were used to determine the virulence level of the strains. The tested strains were adjusted with PBS (1×10^6^ CFU/ml). A group of ten *G. mellonella* larvae was infected with 10 µl of PBS and other groups were injected with 10 μl of bacteria cultures, and incubated at 37°C for 72h to observe the survival of them.

Male BALB/c mice (average approximately 16g, 5 weeks old) were purchased from the Shanghai Medical Laboratory Animal Center (Shanghai, China). Eight mice in each group were infected intravenously with 1 × 10^7^ CFU bacteria. The mortality rate was recorded for 72 h. Animal experiments were repeated twice. Survival curves were generated by Prism 8. Animal ethics approval was obtained from Animal Ethics Committee of Huashan Hospital of Fudan University.

### Siderophores Production Experiment

The relative quantitative siderophores in chelated M9 minimal medium (c-M9) was determined as described previously (Payne, 1994). Briefly, the siderophore assay solution was made by 50 ml of 1.2 mM hexadecyltrimethylammonium bromide, 1.5 ml of 1 mM FeCl_3_ · 6H_2_O, 7.5 ml of 2 mM chrome azurol S (CAS), and 1.37 M piperazine (pH=5.6). 100 ul of each sample was added to wells of a flat-bottom 96-well plate, and then 100 ul of siderophores assay solution with 2% 0.2 M 5-sulfosalicyclic acid was added. The reaction mixture was incubated for 30 min, and were measured at A680 nm. The c-M9 was plus siderophores assay solution plus 5-sulfosalicyclic acid solution was used as a reference (r). The sample (s) should have a lower absorbance than the reference. Siderophore units (Su) are defined as [(Ar - As)/Ar] × 100 = % siderophore units. Each assay was performed in duplicate and repeated three times independently.

Siderophore production was also detected by the CAS agar plate assay (Schwyn and Neilands, 1987). The stationary-phase iron-cheated cultures (3μl) were dropped on CAS plates, and siderophore production was determined by the orange halos after incubation for 48 h at 37°C.

### Statistical Analysis

Prism 8 (GraphPad Software, San Diego, CA) was used to calculate the significance with: unpaired, two-tailed, Student’s *t*-tests, log-rank test; and two-tailed, Mann–Whitney U-test. The type and number of replicates are indicated in the Figure legends.

## Results

### The Conjugative Transfer of pK2606-Like Plasmid Encoding Aerobactin Genes

**Figure 1:**
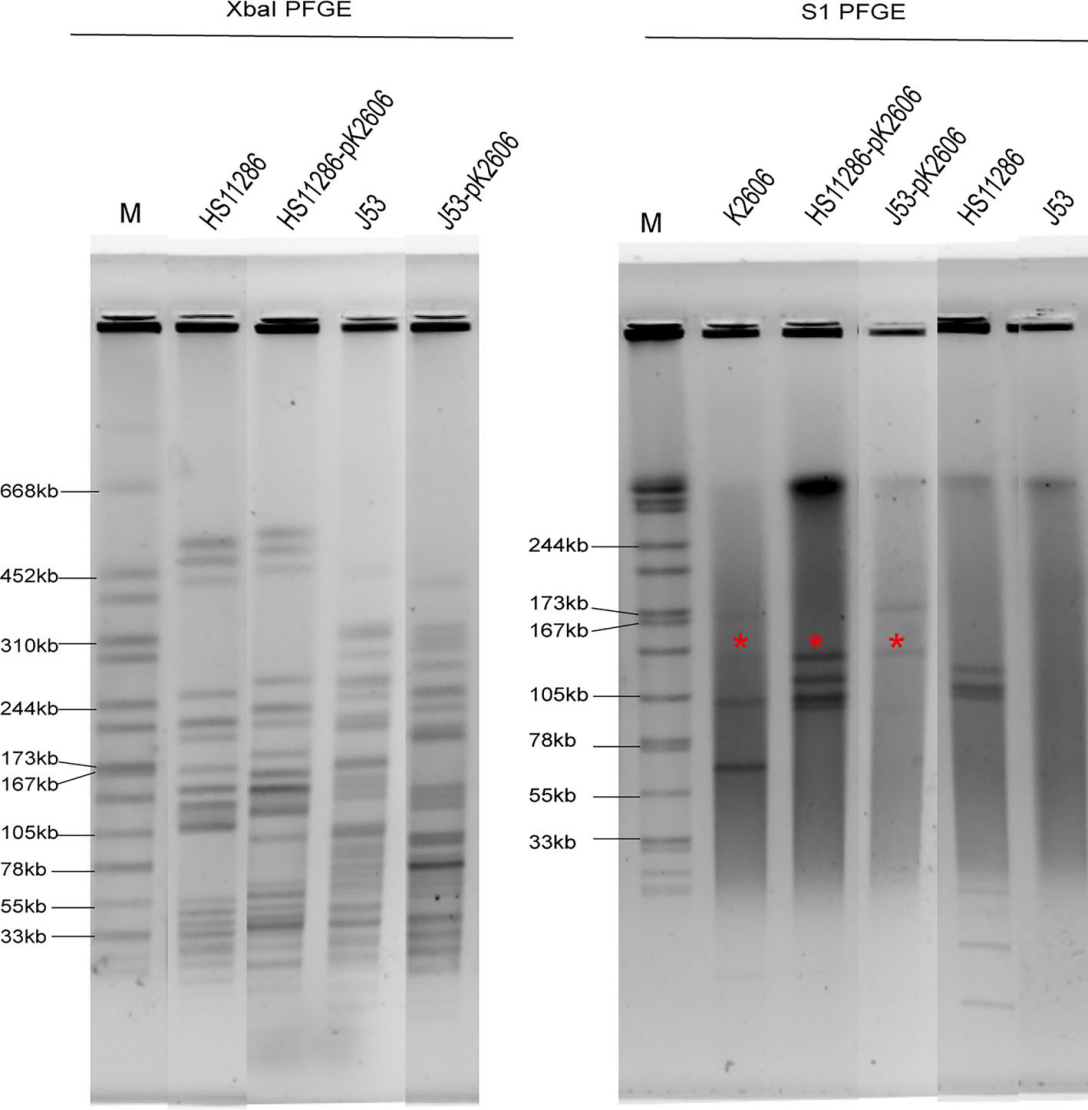
XbaI PFGE and S1-PFGE of strains and corresponding transconjugants. *Virulence plasmid pK2606. The same symbol is used to represent the parental strain and its transconjugant.

K2606 is an ST1027 *Klebsiella pneumoniae* strain, belonging to clonal group 23 (CG23). It harbors 5 plasmids with sizes of 174647bp, 138314bp, 109004bp, 70729bp, and 10077bp, respectively (**Table 1**). The pK2606 (IncFIB_K_/IncFII_K_) plasmid was found to encode aerobactin genes *iucABCD-iutA.* HS11286 is an ST11 carbapenem-resistant *Klebsiella pneumoniae* harboring a KPC plasmid. We found that pK2606 could be conjugated to *Klebsiella pneumoniae* HS11286 as well as *E. coli* J53, and the transconjugant strains HS11286-vir2-pK2606 and J53-vir2-pK2606 were successfully obtained with conjugation frequencies of 2.3×10^-5^ ± 8.9×10^-5^ and 6.4×10^-5^ ± 1.2×10^-6^, respectively. PFGE confirmed the genetic relationship between HS11286 and HS11286-vir2-pK2606, and between J53 and J53-vir2-pK2606 (**Figure 1**). S1-PFGE and draft genome sequencing determined that pK2606 had been transferred to HS11286 and J53 (**Figures 1** and **S1**). Notably, pK2606 was transferred to *E. coli* J53 together with pK2606-1 (**Figure 1**). The KPC plasmid pKPHS2 of *Klebsiella pneumoniae* HS11286 was also self-conjugative. However, we could not obtain the transconjugant of pKPHS2 plasmid transferred to K2606 strain. The *E. coli* J53 strain was susceptible to all tested antimicrobials, while the K2606 strain was susceptible to amikacin, piperacillin/tazobactam, cefepime, chloramphenicol, gentamicin, meropenem, and tigecycline (**Table S1**). Notably, the HS11286 strain was resistant to all tested antimicrobials except tigecycline (**Table S1**).

**Table 1:** Genomic characteristics of K2606, HS11286, and J53.

Strain	MLST	K-type	Chromosome	Plasmids	Resistance determinants	Virulence determinants	Molecular markers
**K2606**	**ST1027**	**KL20**	**5,313,003 bp**	**unamed1**: unidentified, 174647 bp;	**β-lactam**: SHV-27, CTX-M-14; **aminoglycoside**: aadA16,AAC(6’)-Ib-cr6; **fosfomycin**: FosA5; **quinolone**: oqxA, QnrB2, emrR; **trimethoprim**: dfrA27; **sulphonamide**: sul1; **tetracycline**: tet(A);**macrolide**: mphA; **rifamycin**: arr-3.	*iuc*ABCD-*iut*A	*oqxA (+), ICE(-)*
				**unamed2**: IncFIB_K_/IncFII_K_, 138314bp;			
				**unamed3**: IncFIB_pKPSH1_,109004bp;			
				**unamed4**: IncFII_pSE11_, 70729bp;			
				**unamed5**: ColRNAI,10077bp;			
**HS11286**	**ST11**	**KL103**	**5,333,942 bp**	**pKPSH1**: IncFIB_pKPSH1,_ 122799 bp;	**β-lactam**: KPC-2, SHV-11, CTX-M-14, TEM-1; **aminoglycoside**: rmtB, ANT(3’’) , AAC(3), APH(6), APH(3’’); **sulphonamide**: sul2; **tetracycline**: tet(D); **fosfomycin**: FosA6.	None	*oqxA(-), ICE(+)*
				**pKPSH2**: IncFII_K_/IncR, 11195 bp;			
				**pKPSH3**: IncA/C, 105974 bp;			
				**pKPSH4**: Col156, 3751 bp;			
				**pKPSH5**: ColpHAD28, 3353 bp;			
				**pKPSH6**: ColpKPSH6, 1308 bp.			
**J53**	**ST292**	–	**4682574 bp**	None	None	None	*oqxA(-), ICE(-)*

### The Virulence Phenotype of K2606 and Transconjugant Strains

**Figure 2:**
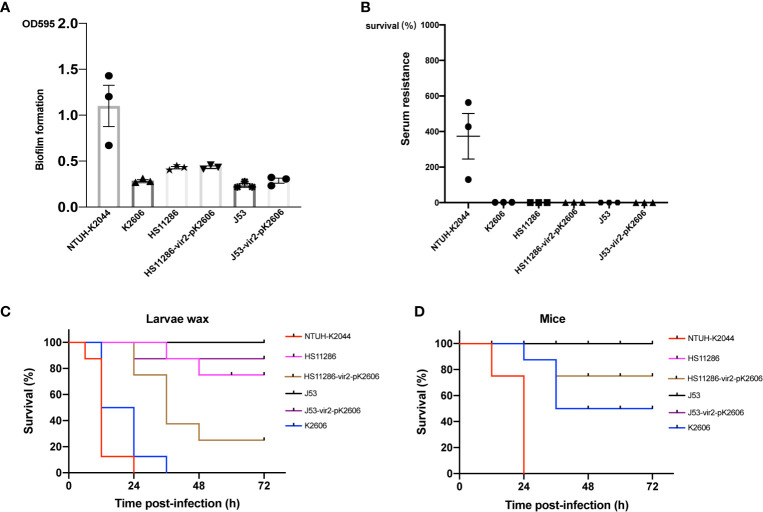
The virulence phenotype of strains and transconjugants. The biofilm formation **(A)** and serum resistance **(B)** of K2606, HS11286, J53, HS11286-vir2-pK2606, and J53-vir2-pK2606. Hypervirulent strain NTUH-K2044 was used as a positive control. An unpaired two-sided Student’s t-test was performed for parental strain and its transconjugant. Each data point was repeated three times (n = 3). Data are presented as the mean ± s.e.m. No significant difference (P>0.05) was observed between HS11286 and HS11286-vir2-pK2606, and between J53 and J53-vir2-pK2606. The virulence level of different strains and their transconjugants as depicted in a larvae wax infection model **(C)** and a mouse infection model **(D)**. Survival of mice (n = 8) infected by each *K. pneumoniae* strain at 72 h is shown. Hypervirulent NTUH-K2044 was used as a positive control. A log-rank (Mantel–Cox) test was performed for the indicated curves. A significant difference (P < 0.0001 in both **C, D**) was observed between HS11286 and HS11286-vir2-pK2606, and between J53 and J53-vir2-pK2606.

Although the K2606 strain harbored the pK2606 plasmid encoding aerobactin, its virulence level still needed to be confirmed. The K2606 strain had a lower biofilm formation ability than hypervirulent NTUH-K2044 (**Figure 2**). Serum resistance experiments showed that the K2606 strain hardly survived in normal healthy serum (**Figure 2**). Although the K2606 strain did not exhibit good biofilm formation capabilities nor serum resistance, it did show a similarly high siderophore production (**Figure 2**), indicating that the *iucABCD-iutA* aerobactin genes exerted their normal function. K2606 strain also showed a significantly higher virulence levels compared to HS11286 and J53 strains in both mice and wax moth larva infection models (**Figure 2**).

**Figure 3:**
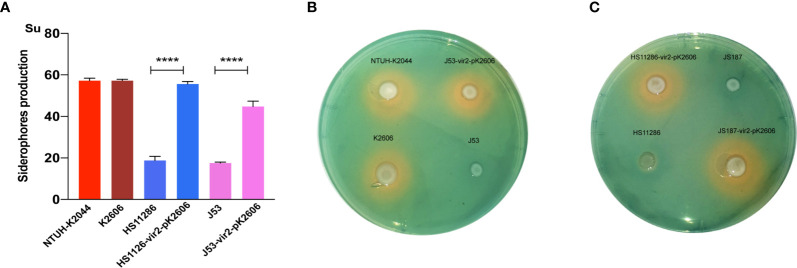
The siderophore production of strains and transconjugants. **(A)** The quantitative siderophore production of K2606, HS11286, J53, HS11286-vir2-pK2606, and J53-vir2-pK2606. An unpaired two-sided Student’s t-test was performed for parental strain and its transconjugant. Each data point was repeated three times (n = 3). Data are presented as the mean ± s.e.m. ****P < 0.0001. **(B, C)** The CAS agar plate assay indicated the siderophore production. Siderophore production was evaluated by the orange halos.

Next, we evaluated whether the acquisition of the pK2606 plasmid could enhance virulence of Klebsiella pneumoniae HS11286 and E. coli J53. The pK2606 plasmid was stable in HS11286 and J53 after 25 passages. Moreover, the acquisition of pK2606 appeared not to affect the growth rate and antimicrobial susceptibility of HS11286-vir2-pK2606 and J53-vir2-pK2606 compared to their parental strains, indicating a very small fitness cost of pK2606 (**Table S1** and **Figure S2**). The biofilm formation and serum resistance of the transconjugants HS11286-vir2-pK2606 and J53-vir2-pK2606 were similar to that of their parental strains (**Figures 2A, B**). However, the acquisition of pK2606 by HS11286 and J53 caused significantly increased siderophore production, reaching the levels of NTUH-K2044 and K2606 (**Figure 3**). Moreover, when mice were infected with 1×10^7^ CFU of bacteria, the virulence level of HS11286-vir2-pK2606 increased, resulting in 25% mortality at 72h, whereas there was 100% survival using the parental HS11286 strain (**Figure 2**). The elevated virulence levels were even more evident using the wax larva infection model. Infection of larvae wax with 1 × 10^6^ CFU of HS11286-vir2-pK2606 led to 25% survival at 72 h, a level much lower than HS11286 (**Figures 2C, D**). Notably, the acquisition of pK2606 plasmid by E. coli J53 appeared not to cause increased virulence. To further confirm our observations, we successfully obtained the transconjugant JS187-vir2-pK2606. The JS187 strain was ST11 carbapenem-resistant Klebsiella pneumoniae which was highly similar to HS11286. Our results showed that JS187-vir2-pK2606 acquired the ability of produce high levels of siderophores and caused high mortality of larvae wax and mice (**Figures S3** and **3C**).

### The Prevalence of pK2606-Like Conjugative Virulence Plasmids in *Klebsiella pneumoniae*

**Figure 4:**
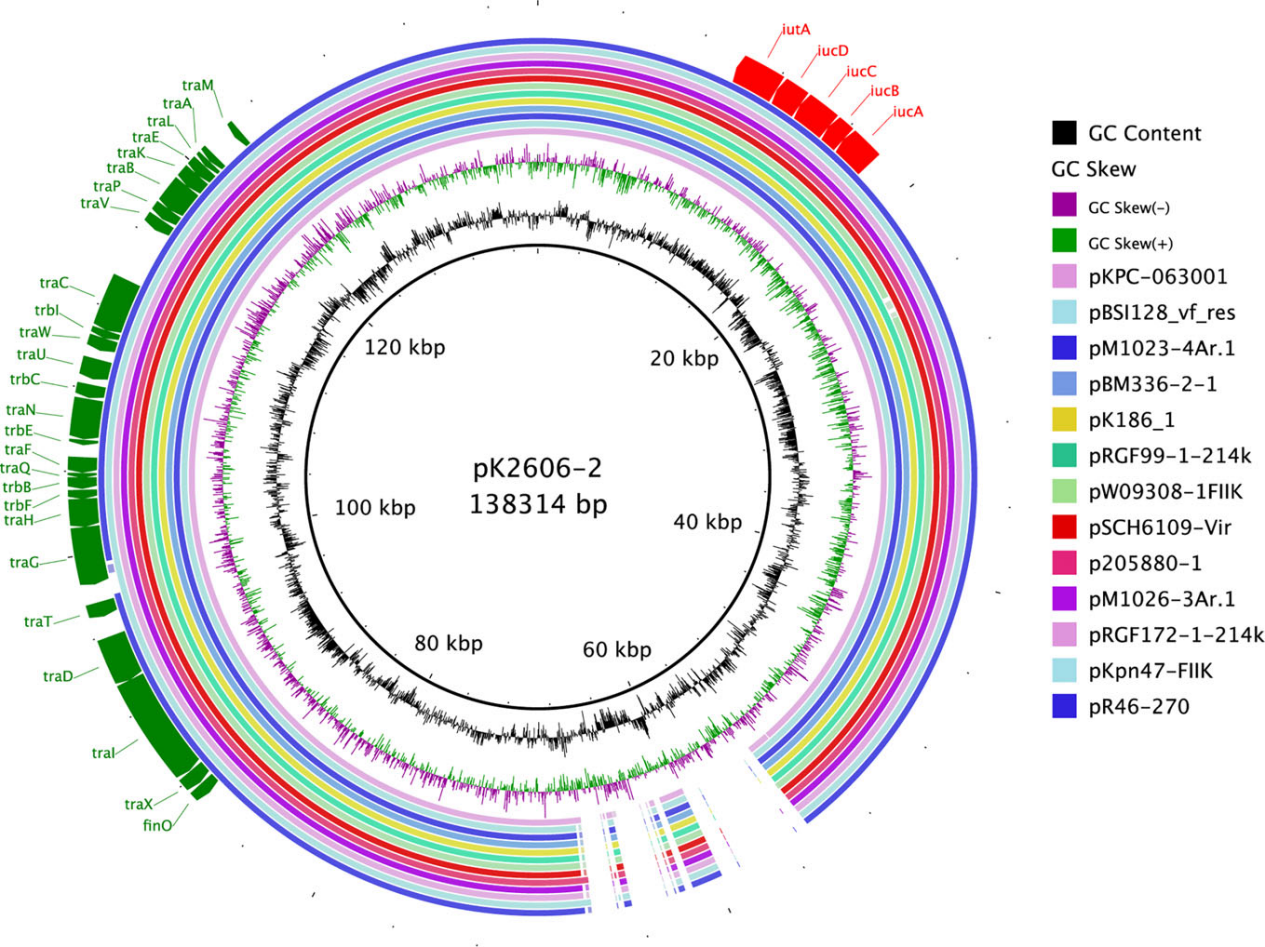
Conjugative pK2606-like virulence plasmids of *Klebsiella pneumoniae* strains in GenBank. Circular maps were generated using BLAST Ring Image Generator (BRIG). pK2606 was used as a reference.

As shown above, pK2606 plasmid was self-conjugative and conferred high siderophore production to recipient strains, thus increasing their virulence. We collected sixteen pK2606-like plasmids from GenBank, which were highly similar to pK2606 and possessed aerobactin genes *iucABCD-iutA* and the T4SS transfer module (**Figure 4**). The K2606 strain was ST1027, belonging to CG23, which is commonly associated with hypervirulent *Klebsiella pneumoniae* strains. Other *Klebsiella pneumoniae* strains isolated from China in 2019 harboring pK2606-like plasmids were ST1, ST36, ST37, ST437, ST290, and ST967, respectively (**Table S2**). The ST distribution and diversity of locations in China suggested such conjugative virulence plasmids may have begun to spread throughout China.

## Discussion

Two possible evolutionary pathways for hypervirulent and carbapenem-resistant *K. pneumoniae* have been proposed: a) the acquisition of a carbapenem-resistance plasmid by K1/K2 hvKP strains, known as CR-hvKp (Hennequin and Robin, 2016) and; b) carbapenem-resistant *K. pneumoniae* (CRKP) acquiring a virulence plasmid, recognized as hv-CRKP (Cejas et al., 2014). The most common virulence plasmids acquired by CRKP strains are non-conjugative pK2044-like plasmids, but their mechanism of transfer is still uncertain. Our previous study identified some conjugative virulence plasmids encoding aerobactin genes in *Klebsiella pneumoniae* (Tian et al., 2021). Considering their potential for self-conjugation, it is essential to confirm the virulence of the host bacteria and their ability to transfer to other strains. CG23 is the most common type of hypervirulent *K. pneumoniae* harboring the pK2044-like virulence plasmid, which contributes to hypervirulence together with its thick capsules (Lam et al., 2018a). We observed a CG23 clinical strain K2606 harboring conjugative plasmid pK2606 encoding aerobactin genes, but no similar pK2044-like plasmids were found. One of the aims of this study was to determine the virulence level of the CG23 K2606 strain and the transfer ability of pK2606.

In comparison to a representative CG23 hypervirulent *Klebsiella pneumoniae* strain, NTUH-K2044, the K2606 strain was not endowed with good biofilm formation ability nor serum resistance. The K2606 strain lacks thick capsules and polysaccharide production and we speculate that thick capsules may largely contribute to biofilm formation and serum resistance. However, high virulence levels of K2606 were still observed in both mice and larvae wax infection models, indicating that high sideroproduction could also strengthen virulence and facilitate survival *in vivo*. This does not preclude that there are other virulence factors in the plasmid possibly influencing the virulence as well. However, capsular and siderophore production are always considered as two key factors contributing to the pathogenicity of hvKp strains (Russo and Marr, 2019). Efficient iron acquisition is necessary for hvKp to survive in human ascites. Thus, if the hvKp strain could produce more iron acquisition factors, it may acquire increased virulence. However, determining, whether pK2606 carrying *iucABCD-iutA* confers virulence to other strains is necessary in order to prevent the dissemination of conjugative pK2606-like virulence plasmids.

Conjugative virulence plasmids were possibly formed by the insertion of an aerobactin cluster into an IncFIB_K_/IncFII conjugative plasmid (Tian et al., 2021), and the composition of pK2606 is concordant with this observation. There are also some other reports concerning conjugative virulence plasmids. A previous study indicated that the virulence plasmid p15WZ-82_Vir with a T4SS in *K. variicola* could be transferred to a CRKP strain and *E. coli* (Yang et al., 2019). Li et al. also reported the conjugative virulence plasmid p17-15-vir in *Klebsiella pneumoniae* ST15 (Li et al., 2020). The two large hybrid virulence plasmids p15WZ-82_Vir (282Kb) and p17-15-vir (479Kb) were possibly unstable and brought potential fitness costs to their host bacteria. In comparison, pK2606 is just 138 Kb and possesses a complete Type IV Secretion System with an encoded aerobactin cluster. Notably, pK2606 could efficiently transfer to ST11 CRKP strains and also *E. coli* J53, conferring high siderophore production abilities and facilitating their survival *in vivo*. It has long been known that bacteria require iron for growth and that their ability to acquire iron is requisite for growth and survival (Page, 2019). Therefore, most bacteria can produce diverse siderophores, including aerobactin, enterobactin, salmochelin, and yersiniabactin, to acquire iron in iron-depleted environments, such as the human host. Aerobactin is a critical virulence factor for hvKp, while the additional siderophores are not major contributors to high siderophore levels (Russo et al., 2015). The virulence plasmids acquired by ST11 CRKP strains always display deleted salmochelin coding genes, which would not affect their ability to confer hypervirulence to host bacteria (Gu et al., 2018). The pK2606 plasmid encodes only aerobactin, and the acquisition of pK2606 by HS11286 and J53 caused significantly increased siderophore production, reaching similarly high levels to the hypervirulent NTUH-K2044. We observed that the transconjugant HS11286-vir2-pK2606 and J53-vir2-pK2606 were not only endowed with high siderophore levels, but also high virulence levels in mice and larvae wax infection models.

Our study confirmed the hypervirulence of the K2606 strain and efficient transfer of the pK2606 plasmid, which could confer virulence to other strains. There are some indications that the conjugative virulence plasmids have been disseminated, for example, pK2606-like conjugative virulence plasmids were identified amongst various STs of *Klebsiella pneumoniae* strains. CG23 strains do not always carry classical pK2044-like virulence plasmids, and disturbingly, the pK2606-like conjugative virulence plasmids can be successfully transferred to CRKP strains to further evolve into hv-CRKP. Conjugative virulence plasmids almost always carry aerobactin genes, while the mucoid regulator *rmpA* was not found, thus, may not confer the hypermucoviscous phenotype. However, plasmid-encoded aerobactin appears sufficient to facilitate host bacterial survival and severe infections *in vivo* (Russo et al., 2015). Nevertheless, the acquisition of pK2606-like conjugative virulence plasmids may not produce as high a virulence phenotype as non-conjugative pK2044-like plasmids, but the consequences of their conjugal transfer are relatively serious, especially transference to multi-drug resistant strains. The deficiency of host immune defense systems in ST11 CRKP strains facilitates the invasion of such conjugative virulence plasmids (Tang et al., 2020; Zhou et al., 2020). Thus, prompt action is needed with specific interventions to prevent another global epidemic like the KPC plasmids。

In conclusion, this study confirmed the hypervirulence phenotype of *Klebsiella pneumoniae* strain K2606 is caused by high siderophore levels. Additionally, we found that pK2606-like conjugative virulence plasmids could confer virulence to other strains. The pK2606-like virulence plasmids are self-conjugative and have emerged in various *Klebsiella pneumoniae* strains, including multi-drug resistant strains. Clinicians and researchers should be vigilant for the emergence of hv-CRKP strains caused by the transmission of such conjugative virulence plasmids in the clinical setting.

## Data Availability Statement

The datasets presented in this study can be found in online repositories. The names of the repository/repositories and accession number(s) can be found in the article/**Supplementary Material**.

## Ethics Statement

The animal study was reviewed and approved by Animal Ethics Committee of Huashan Hospital of Fudan University.

## Author Contributions

All the authors listed have made a substantial, direct and intellectual contribution to this work, and approved the submitted version for publication.

## Funding

This work was supported by Science and Technology Commission of Shanghai Municipality under Grant 19JC1413002, National Natural Science Foundation of China under Grant 81871692 and 82172315, Shanghai Municipal Key Clinical Specialty under grant shslczdzk0330, and Jiaxing Municipal Key Laboratory of Infectious Diseases and Bacterial Drug Resistance.

## Conflict of Interest

The authors declare that the research was conducted in the absence of any commercial or financial relationships that could be construed as a potential conflict of interest.

## Publisher’s Note

All claims expressed in this article are solely those of the authors and do not necessarily represent those of their affiliated organizations, or those of the publisher, the editors and the reviewers. Any product that may be evaluated in this article, or claim that may be made by its manufacturer, is not guaranteed or endorsed by the publisher.
