# Supplementary material for: Acquisition of the Conjugative Virulence Plasmid From a CG23 Hypervirulent Klebsiella pneumoniae Strain Enhances Bacterial Virulence
Source: Front Cell Infect Microbiol. 2021 Sep 9;11:752011. doi: 10.3389/fcimb.2021.752011 (PMC8485780; doi:10.3389/fcimb.2021.752011)
Supplement: Supplementary file 1 [file DataSheet_1.pdf]

Acquisition of the conjugative virulence plasmid from a CG23 hypervirulent *Klebsiella pneumoniae* strain enhances bacterial virulence

SUPPLEMENTARY DATA

Figure S1. MUMmer-based comparison of plasmids of transconjugants and referenced plasmids. (A) Comparison of plasmids of the transconjugant HS11286-vir2-pK2606 and referenced plasmids of HS11286 and K2606 strains. (B) Comparison of plasmids of the transconjugant J53-vir2-pK2606 and referenced plasmids of K2606 strains. The abscissa indicates the plasmid contigs of transconjugants and the ordinate the reference plasmids.

Figure S2. Growth curve of strains and transconjugants. (A) Growth curve of HS11286 and HS11286-vir2-pK2606. (B) Growth curve of J53 and J53-vir2-pK2606. y axis, optical densities (OD) of broth cultures at 600 nm; x axis, time of growth (hours).

Figure S3. Siderophore production and virulence levels of JS187 and JS187-vir2-pK2606. (A) Quantitative siderophore production of JS187 and JS187-vir2-pK2606. A significance of siderophore production of JS187 and JS187-vir2-pK2606 was observed (****$P < 0.0001$). An unpaired two-sided Student's t-test was performed. Each data point was repeated three times ($n = 3$). The virulence level of JS187 and JS187-vir2-pK2606 as depicted in a larvae wax infection model (B) and a mouse infection model (C). Survival of mice ($n = 8$) infected by each *K. pneumoniae* strain at 72 h is shown. Hypervirulent NTUH-K2044 was used as a positive control. A log-rank (Mantel–Cox) test was performed for the indicated curves. A significant difference ($P < 0.001$ in both B and C) was observed between JS187 and JS187-vir2-pK2606.

Table S1. Antimicrobial susceptibilities of K2606, HS11286, J53, and their transconjugants.

Table S2. The characteristics of pK2606-like conjugative virulence plasmids.

Table S3. Primers used in this study.

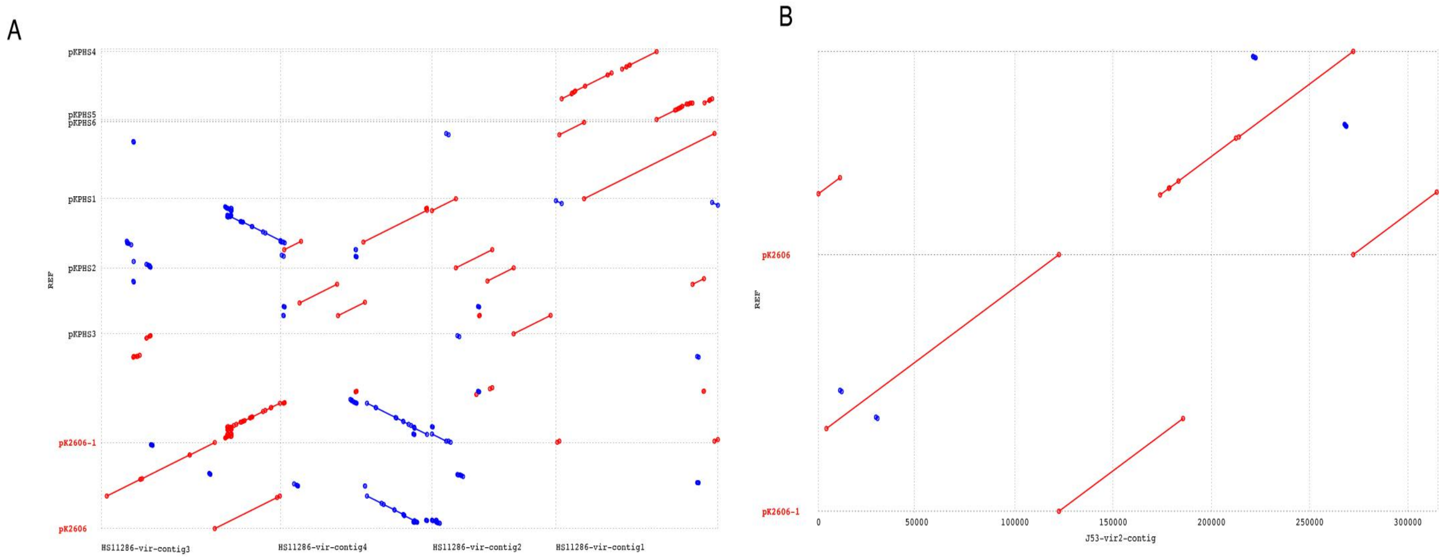

Figure S1. MUMmer-based comparison of plasmids of transconjugants and referenced plasmids. The abscissa indicates the plasmid contigs of transconjugants and the ordinate the reference plasmids.

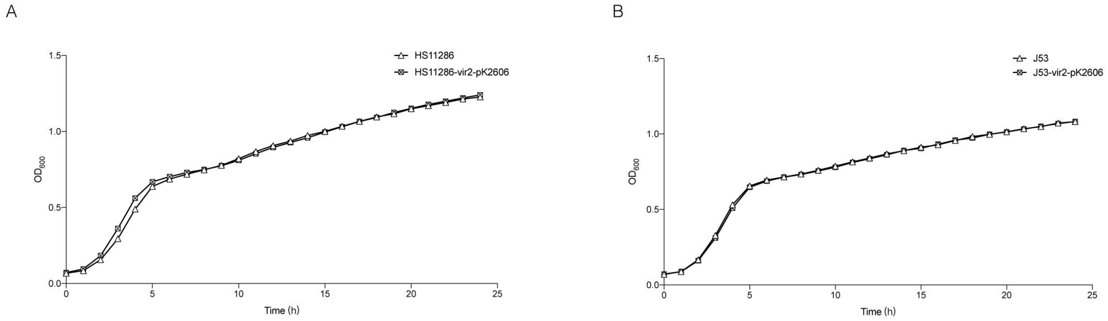

Figure S2. Growth curve of strains and transconjugants. y axis, optical densities (OD) of broth cultures at 600 nm; x axis, time of growth (hours).

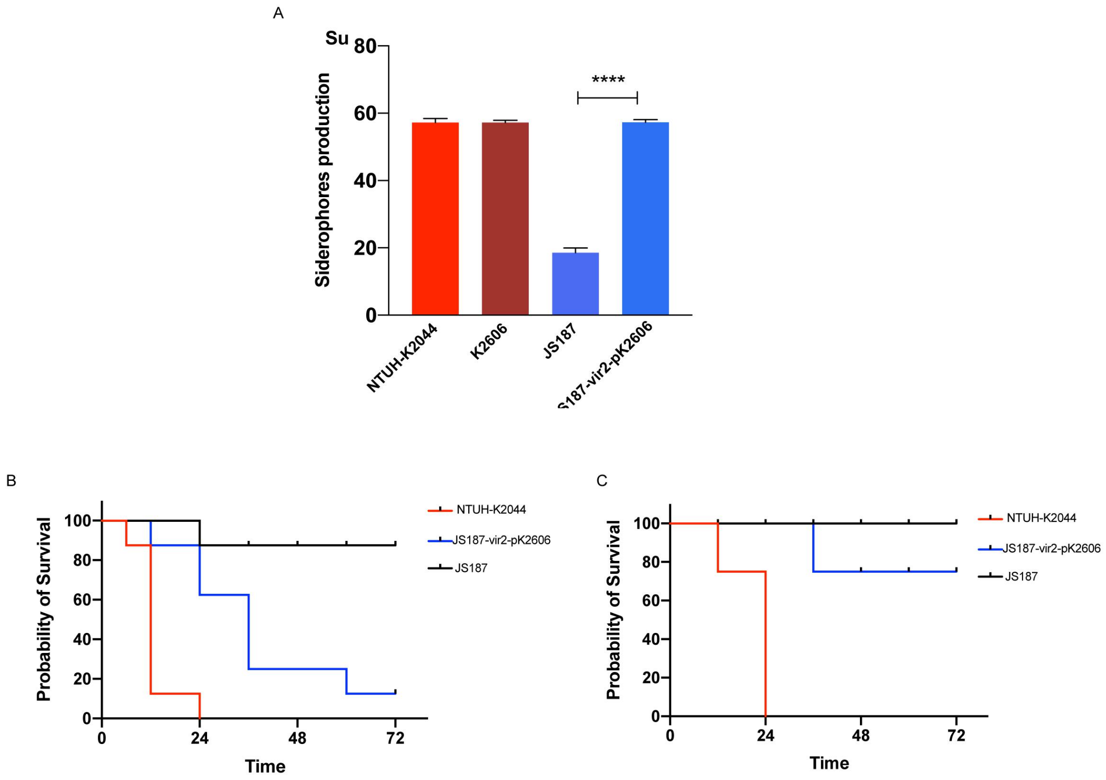

Figure S3. Siderophore production and virulence levels of JS187 and JS187-vir2-pK2606. A significance of siderophore production of JS187 and JS187-vir2-pK2606 was observed (***P < 0.0001). An unpaired two-sided Student's t-test was performed. Each data point was repeated three times (n = 3).

Table S1. Antimicrobial susceptibilities of K2606, HS11286, J53, and their transconjugants

Strains	STs	bacterial species	MIC (μg ml^{-1})[a]													
			AMK	SAM	TZP	CZO	CXM	CRO	FEP	CHL	GEN	LVX	SXT	MEM	TGC	NIT
K2606	ST23	*K. pneumoniae*	<8	32/16	<8/4	>16	>64	>8	2	8	<1	2	>8/152	<0.25	1	128
HS11286	ST11	*K. pneumoniae*	>128	>64/32	>256/4	>16	>64	>8	16	>64	>32	2	>8/152	64	<0.5	256
JS187	ST11	*K. pneumoniae*	>128	>64/32	>256/4	>16	>64	>8	>64	>64	>32	8	>8/152	32	<0.5	256
J53	-	*E. coli*	<8	4/2	<8/4	1	8	<0.25	<2	8	<1	<0.5	<0.25/4.75	<0.25	<0.5	<8
**Transconjugants**																
HS11286-vir2-pK2606	ST11	*K. pneumoniae*	>128	>64/32	>256/4	>16	>64	>8	16	>64	>32	8	>8/152	64	<0.5	128
JS187-vir2-pK2606	ST11	*K. pneumoniae*	>128	>64/32	>256/4	>16	>64	>8	>64	>64	>32	16	>8/152	32	1	>256
J53-vir2-pK2606	-	*K. pneumoniae*	<8	8/4	<8/4	2	4	<0.25	<2	8	2	<0.5	<0.25/4.75	<0.25	<0.5	<8

[a]AMK, amikacin; SAM, ampicillin/sulbactam; TZP, piperacillin/tazobactam; CZO, cefazolin、CXM, cefuroxime; CRO, ceftriaxone; FEP, cefepime; CHL, chloramphenicol; GEN, gentamicin; LVX, levofloxacin; SXT, trimethoprim/sulphamethoxazole; MEM, meropenem; TGC, tigecycline; NIT, nitrofurantion.

Table S2. The characteristics of pK2606-like conjugative virulence plasmids.

Plasmids	Accession number	Strain	Chromosome	MLST	Isolated Year	Isolated contries
p205880-1	CP030303.1	205880	CP030302.1	290	2012	China：Beijing
pSCH6109-Vir	CP050860.1	SCH6109	CP050858.1	37	2016	China: Shanghai
pM1023-4Ar.1	CP063852.1	M1023-4Ar	CP063851.1	1	2019	China: Baoding
pM1026-3Ar.1	CP063859.1	M1026-3Ar	CP063858.1	1	2019	China: Baoding
pBM336-2-1	CP063914.1	BM336-2-1	CP063913.1	36	2019	China: Tangshan
pfekpn2511-1	CP068973.1	fekpn2511	CP068972.1	290	2019	China: Hengyang
pRGF172-1-214k	CP075277.1	RGF172-1	CP075275.1	290	2019	China: Nantong
pRGF99-1-214k	CP075553.1	RGF99-1	CP075552.1	967	2019	China: Nantong
pK186_1	CP076519.1	K186	CP076518.1	437	2017	China: Hangzhou
pR46-270	CP035776.1	R46	CP035777.1	3796	2015	China: Wenzhou
pBSI128_vf_res	MT269849.1	BSI128	-	-	-	-
pKPC-063001	MZ156798.1	KP63	-	-	-	-
p130411-38618_1	MK649826.1	130411-38618	-	-	-	-
pW09308-1FIIK	MN821363.1	9308	-	-	-	-
pKpn47-FIIK	MN821369.1	Kpn47	-	-	-	-

Table S3. Primers used in this study

Name	Sequences (5'-3')
For constructing of pK2606-kana	
Up-F	CTGACTGGTTACTCCGGCAA
Up-R	cctacacaatcgctcaagacgtACCTGTCATCCATACTGCGT
Kana-F	ACGTCTTGAGCGATTGTGTAGG
Kana-R	AGCCATGGTCCATATGAATATCCTC
Down-F	gaggatattcatatggaccatggctAGCAGCAGGGTCGCATAATA
Down-R	TCAGTCGTTTTCGTGCCGTT
For screening transconjugants	
iucA-F	GCTTATTTCTCCCCAACCC
iucA-R	TCAGCCCTTTAGCGACAAG
KPC-F	CTGTCTTGTCTCTCATGGCC
KPC-R	CCTCGCTGTGCTTGTCATCC
oqxA-F	CCAAAGTGACCGCCCCTATT
oqxA-R	GACGATGACGCTATCCCCAG
ICE-F	GGTGAGCTGACGAATGGATT
ICE-R	TTTCTCGGCACCAACTCAAA
